# Exploring international differences in ovarian cancer care: a survey report on global patterns of care, current practices, and barriers

**DOI:** 10.1136/ijgc-2023-004563

**Published:** 2023-08-17

**Authors:** Marc Daniël Algera, Rhett Morton, Sudha S Sundar, Rhonda Farrell, Willemien J van Driel, Donal Brennan, Marcus J Rijken, Selina Sfeir, Lucy Allen, Mary Eiken, Robert L Coleman, the collaborators of the Global Equality in Ovarian Cancer Care project group, A Alvarez Secord

**Affiliations:** 1 Gynecology Oncology, Maastricht UMC+, Maastricht, The Netherlands; 2 Scientific Bureau, Dutch Institute for Clinical Auditing, Leiden, The Netherlands; 3 GROW- School for Oncology and Reproduction, Maastricht, The Netherlands; 4 Obstetrics and Gynaecology, Royal Prince Alfred Hospital, Camperdown, New South Wales, Australia; 5 Department of Gynaecology Oncology, University of Birmingham, West Midlands, UK; 6 Gynaecology, Royal Hospital for Women, Sydney, New South Wales, Australia; 7 Gynecologic Oncology, The Netherlands Cancer Institute - Antoni van Leeuwenhoek Hospital, Amsterdam, The Netherlands; 8 Gynaecology Oncology, Mater Misericordiae University Hospital, Dublin, Ireland; 9 Department of Gynaecology Oncology, University of Birmingham, Birmingham, UK; 10 International Gynecologic Cancer Society, Austin, Texas, USA; 11 Sarah Cannon Research Institute, Nashville, Tennessee, USA

**Keywords:** carcinoma, ovarian epithelial, cytoreduction surgical procedures

## Abstract

**Objective:**

Although global disparities in survival rates for patients with ovarian cancer have been described, variation in care has not been assessed globally. This study aimed to evaluate global ovarian cancer care and barriers to care.

**Methods:**

A survey was developed by international ovarian cancer specialists and was distributed through networks and organizational partners of the International Gynecologic Cancer Society, the Society of Gynecologic Oncology, and the European Society of Gynecological Oncology. Respondents received questions about care organization. Outcomes were stratified by World Bank Income category and analyzed using descriptive statistics and logistic regressions.

**Results:**

A total of 1059 responses were received from 115 countries. Respondents were gynecological cancer surgeons (83%, n=887), obstetricians/gynecologists (8%, n=80), and other specialists (9%, n=92). Income category breakdown was as follows: high-income countries (46%), upper-middle-income countries (29%), and lower-middle/low-income countries (25%). Variation in care organization was observed across income categories. Respondents from lower-middle/low-income countries reported significantly less frequently that extensive resections were routinely performed during cytoreductive surgery. Furthermore, these countries had significantly fewer regional networks, cancer registries, quality registries, and patient advocacy groups. However, there is also scope for improvement in these components in upper-middle/high-income countries. The main barriers to optimal care for the entire group were patient co-morbidities, advanced presentation, and social factors (travel distance, support systems). High-income respondents stated that the main barriers were lack of surgical time/staff and patient preferences. Middle/low-income respondents additionally experienced treatment costs and lack of access to radiology/pathology/genetic services as main barriers. Lack of access to systemic agents was reported by one-third of lower-middle/low-income respondents.

**Conclusions:**

The current survey report highlights global disparities in the organization of ovarian cancer care. The main barriers to optimal care are experienced across all income categories, while additional barriers are specific to income levels. Taking action is crucial to improve global care and strive towards diminishing survival disparities and closing the care gap.

**WHAT IS ALREADY KNOWN ON THIS TOPIC**

- Global disparities in ovarian cancer survival rates exist. However, the variation in care has only been assessed in certain high-income countries. Therefore, until now, no recommendations could be made on improving global ovarian cancer care, particularly in middle- and low-income countries.

**WHAT THIS STUDY ADDS**

- The current study describes the first survey report on global ovarian cancer care organization, including data from high-, middle-, and low-income countries. Data were provided by over 1000 ovarian cancer specialists from 115 countries, which makes the current study the most extensive expert opinion survey study in gynecological oncology. Disparities in care organization were observed across income categories, revealing opportunities to improve care. Main barriers to optimal care for the entire group were patient co-morbidities, advanced presentation, and social factors such as travel distance and support systems. Besides, income category-specific barriers were identified, giving a unique insight into the experienced barriers at a regional level.

**HOW THIS STUDY MIGHT AFFECT RESEARCH, PRACTICE OR POLICY**

- Additional analyses should be performed on the current data with regional representatives to enable country-specific recommendations on improving care globally. These country-specific recommendations should be implemented to improve overall quality and equity in ovarian cancer care and outcomes.

## Introduction

Ovarian cancer has the highest mortality rate of all gynecological malignancies worldwide.^1^ Unfortunately, substantial global disparities in survival rates exist for patients with ovarian cancer, whereby those from resource-poor countries frequently have poorer prognoses; however, differences in survival rates also exist across upper-middle and high-income countries.^2–8^

The underlying causes for these survival disparities are multifactorial. According to data from seven high-income countries, the variation in survival could partly be explained by the differences in stage at diagnosis. However, international survival disparities were also found within each stage, suggesting unequal access to optimal treatment in certain high-income countries.^5 6^

Local guideline variation and whether patients are treated according to international guidelines could give insight into why patients receive sub-optimal treatment globally. Recently, a detailed analysis of guidelines and care organization was performed with data from certain high-income countries.^7^ Patterns of care varied substantially, mostly in primary versus interval cytoreductive surgery rates, willingness to undertake extensive surgery (correlated with survival), and perceived barriers to optimal cytoreduction. These results give a unique insight into the variation in care in high-income countries, but lack data from middle/low-income countries where the disparities are probably even more considerable.

Health system barriers could also result in international variation in survival rates for patients with ovarian cancer. Lack of adequate hospital staffing and lack of treatment monitoring via audits were described as the most important factors that negatively influenced optimal care by physicians in high-income countries. Moreover, access to systemic therapies was also described as a barrier.^7^ Presumably, physicians in resource-poor countries face these and other barriers; however, these physicians were not included in previous studies.

Racial and socioeconomic differences within individual countries could also lead to survival inequalities for patients with ovarian cancer.^8–14^ In a study from the USA, African-American women had lower survival rates than white women with ovarian cancer. The possible cause given was that African-American women do not always receive appropriate guideline-based treatment because of health insurance and other socioeconomic reasons.^8^ Whether these causes for survival disparities exist within other countries around the globe is plausible but as yet is unclear.

The current literature gives an insight into the disparities in ovarian cancer survival; however, it lacks a global assessment of the patterns of—and barriers to—optimal care, particularly in middle/low-income countries. Therefore, the International Gynecologic Cancer Society has initiated a project to evaluate and address ovarian cancer care at a global level: Global Equality in Ovarian Cancer Care. This project aims to ultimately achieve equality and equity in healthcare for all women with ovarian cancer, in line with the slogan of World Cancer Day 2023 “Close the care gap”.^15^ As the first step of this project, an expert opinion survey was developed. The current report describes the structure and first results.

## Methods

### Developing the Survey

International experts in ovarian cancer care developed the survey. First, the aims were clarified and survey questions were constructed through four online meetings. Then, after reviewing multiple questions, a consensus was reached on a draft survey. Furthermore, the project group reached out to a larger group of experts to review the selected questions. After revision, the survey consisted of 30 questions divided into three sections: (1) respondent characteristics; (2) national/regional healthcare organization; and (3) individual hospital healthcare organization (see Online supplemental table 1).

10.1136/ijgc-2023-004563.supp1 Supplementary data

### Distribution of the Survey

The survey was distributed through the networks of (the strategic alliance partners of) the International Gynecologic Cancer Society, the Society of Gynecologic Oncology, and the European Society of Gynecological Oncology. Therefore, the project reached a worldwide network of physicians treating ovarian cancer. Furthermore, specialists from countries not represented in the networks were targeted. The survey was distributed in English, Spanish, Portuguese, Mandarin, and Russian.

### Statistical Analysis

The respondents were stratified by World Bank Income category (high-income, upper-middle income, and lower-middle/low-income). The associations between categorical data and income category were analyzed using univariable logistic regressions. Income was selected as an independent variable, and the categorical survey outcomes were selected as dependent variables. Multi-level logistic regressions (random intercept) were performed to adjust for clustering effects within countries.^16^ Descriptive statistics were also used. Data were analyzed using RStudio 1.4.1106 (RStudio, USA, 2021).

## Results

### Respondent Characteristics

**Figure 1:**
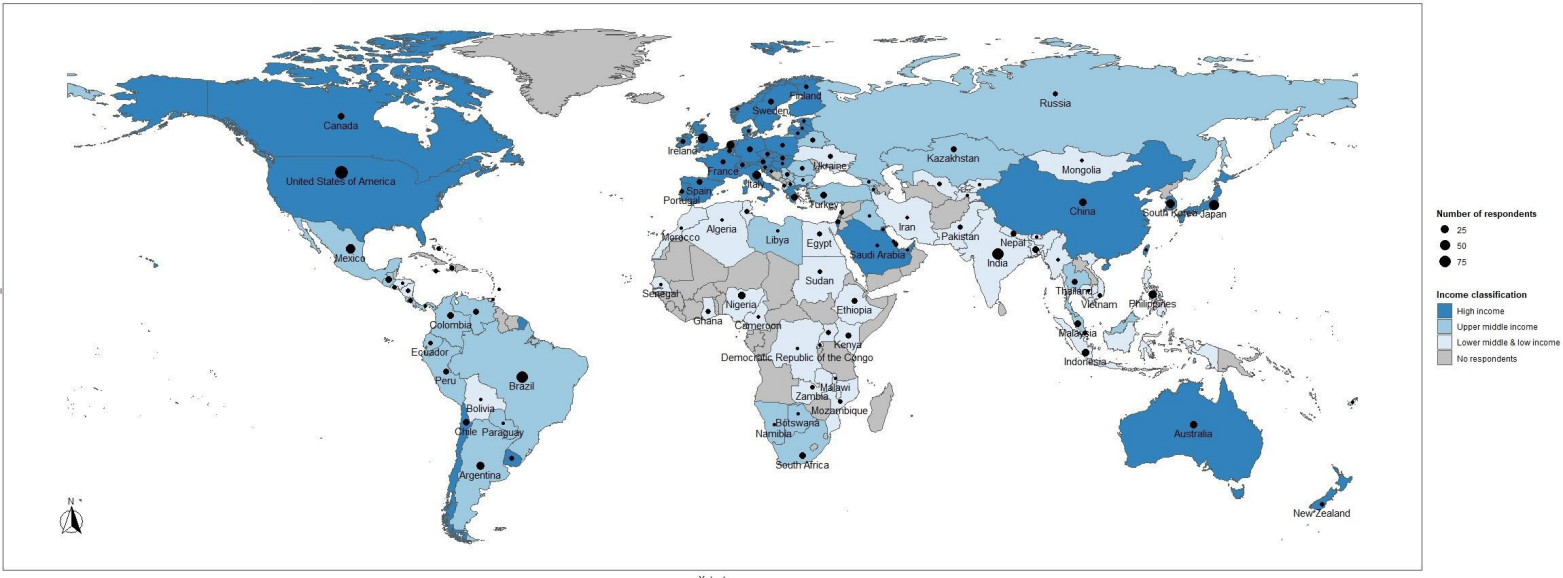
Global Equality in Ovarian Cancer Care Survey: number of responses per country.

A total of 1059 physicians from 115 countries completed the survey (Figure 1). The three countries with the most responses were the USA (n=98), Brazil (n=76), and India (n=74). The number of respondents per country (and continent) are shown in Online supplemental table 2. Most respondents were (subspecialty-accredited) gynecological cancer surgeons (83%, n=887), some of whom also provide systemic treatment. Obstetricians/gynecologists represented 8% of the respondents (n=80) and other specialists represented the remaining 9% (n=92: medical oncologists, radiation oncologists, pathologists, nuclear medicine specialists, hematologist-oncologist, genetics professional, pelvic surgeon). World Bank Income category breakdown was as follows: high-income countries (46%, n=486), upper-middle-income countries (29%, n=303), and lower-middle/low-income countries (25%, n=270).

### Organization of National/Regional Healthcare

#### Guidelines

Almost all respondents used guidelines from the National Comprehensive Cancer Network, European Society of Gynecological Oncology, and/or European Society for Medical Oncology (n=1023). Some respondents (n=29) stated they used other international guidelines (International Federation of Gynecology and Obstetrics and other organizations). Moreover, national/regional guidelines were used next to the previously mentioned guidelines by two-thirds of the respondents (n=696). These national/regional guidelines were often derived from the previously mentioned international guidelines. Seven respondents used no guidelines. Most respondents stated that >75% of their patients were treated according to (inter)national guidelines (n=841).

#### Cancer Registries, Quality Registries, and Patient Advocacy Groups

Responses from lower-middle/low-income countries were associated with fewer cancer registries, quality registries, and patient advocacy groups in multi-level univariable logistic regressions (Table 1). Moreover, responses from upper-middle-income countries were associated with fewer patient advocacy groups. While 86% of the respondents from high-income countries stated their institution contributed to a cancer registry, only 62% also contributed to a quality registry. Also, nearly one-third of the high-income respondents stated that no patient advocacy group was active in their country.

**Table 1:** Univariable and multi-level logistic regression analyses showing the associations between income classification and cancer registries, quality registries, patient advocacy groups, cancer centers, regional networks, and surgical training programs

				Univariable logistic regression analysis			Multi-level analysis corrected for country		
		**Cancer registry**							
**Income classification**	**Total**	**Yes**	**No**	**OR**	**95% CI**	**P value**	**OR**	**95% CI**	**P value**
High	486	417 (85.8)	69 (14.2)	1			1		
Upper-middle	303	234 (77.2)	69 (22.8)	0.6	0.4 to 0.8	0.002	1	0.4 to 2.2	0.938
Low or lower-middle	270	190 (70.4)	80 (29.6)	0.4	0.3 to 0.6	<0.001	0.4	0.2 to 0.8	0.007
		**Quality registry**							
**Income classification**	**Total**	**Yes**	**No**	**OR**	**95% CI**	**P value**	**OR**	**95% CI**	**P value**
High	486	299 (61.5)	187 (38.5)	1			1		
Upper-middle	303	147 (48.5)	156 (51.5)	0.6	0.4 to 0.8	<0.001	1,0	0.5 to 1.7	0.879
Low or lower-middle	270	128 (47.4)	142 (52.6)	0.6	0.4 to 0.8	<0.001	0.6	0.4 to 1.1	0.087
		**Patient advocacy**							
**Income classification**	**Total**	**Yes**	**No**	**OR**	**95% CI**	**P value**	**OR**	**95% CI**	**P value**
High	486	344 (70.8)	142 (29.2)	1			1		
Upper-middle	303	109 (36.0)	194 (64.0)	0.2	0.2 to 0.3	<0.001	0.2	0.1 to 0.4	<0.001
Low or lower-middle	270	68 (25.2)	202 (74.8)	0.1	0.1 to 0.2	<0.001	0.1	0.1 to 0.3	<0.001
		**Cancer center**							
**Income classification**	**Total**	**Yes**	**No**	**OR**	**95% CI**	**P value**	**OR**	**95% CI**	**P value**
High	486	314 (64.6)	172 (35.4)	1			1		
Upper-middle	303	182 (60.1)	121 (39.9)	0.8	0.6 to 1.1	0.199	1	0.5 to 2.0	0.977
Low or lower-middle	270	144 (53.3)	126 (46.7)	0.6	0.5 to 0.8	<0.001	0.5	0.3 to 1.0	0.052
		**Regional networks**							
**Income classification**	**Total**	**Yes**	**No**	**OR**	**95% CI**	**P value**	**OR**	**95% CI**	**P value**
High	486	206 (42.4)	280 (57.6)	1			1		
Upper-middle	303	89 (29.4)	214 (70.6)	0.6	0.4 to 0.8	<0.001	0.4	0.2 to 0.8	0.013
Low or lower-middle	270	45 (16.7)	225 (83.3)	0.3	0.2 to 0.4	<0.001	0.2	0.1 to 0.5	<0.001
		**Surgical training program**							
**Income classification**	**Total**	**Yes**	**No**	**OR**	**95% CI**	**P value**	**OR**	**95% CI**	**P value**
High	486	425 (87.4)	61 (12.6)	1			1		
Upper-middle	303	273 (90.1)	30 (9.9)	1.3	0.8 to 2.1	0.258	1.3	0.5 to 3.5	0.615
Low or lower-middle	270	248 (91.9)	22 (8.1)	1.6	1.0 to 2.8	0.066	1.4	0.5 to 3.5	0.532

#### Cancer Centers, Regional Networks, and Surgical Training Programs

Almost 40% of the respondents stated there was no cancer center for ovarian cancer, and most respondents stated that no regional networks existed in their countries (Table 1). Respondents working in middle/low-income countries were significantly less likely to have established regional networks, multi-disciplinary teams, referral pathways, and agreed locations of surgical procedures (Table 1). The implementation of surgical training programs was similar between income categories (Table 1).

### Organization of Healthcare in Individual Hospitals

#### Type of Surgeon

In the entire group, gynecological cancer surgeons performed most operations. Almost 20% of the respondents from lower-middle/low-income countries answered that obstetricians/gynecologists performed cytoreductive surgeries (data not shown).

#### Multi-disciplinary Team Meetings

Most respondents held multi-disciplinary team meetings. However, the odds of multi-disciplinary team meetings in individual hospitals significantly decreased in upper-middle and lower-middle/low-income countries (Table 2A).

**Table 2:** Survey results of the questions regarding multi-disciplinary team meetings

A: Univariable and multi-level logistic regression analyses showing the associations between income classification and survey responses on whether multi-disciplinary team meetings are standard of care									
		Multi-disciplinary team meetings		Univariable logistic regression analysis			Multi-level analysis corrected for country		
Income classification	Total	Yes	No	OR	95% CI	P value	OR	95% CI	P value
High	486	459 (94.4)	27 (5.6)	1			1		
Upper-middle	303	265 (87.5)	38 (12.5)	0.4	0.2 to 0.7	<0.001	0.4	0.2 to 0.8	0.017
Low or lower-middle	270	243 (90.0)	27 (10.0)	0.5	0.3 to 0.9	0.025	0.4	0.2 to 0.8	0.01

B: Members of multi-disciplinary team according to the respondents of the survey					
Multi-disciplinary team members	High-income Yes, n (%)	Upper-middle income Yes, n (%)	Lower-middle and low-income Yes, n (%)	Total Yes, n (%)	P value (χ^2^)
Gynecological oncologist	477 (98.1)	262 (86.5)	231 (85.6)	970 (91.6)	<0.01
Medical oncologist	344 (70.8)	263 (86.8)	208 (77.0)	815 (76.9)	<0.01
Surgical oncologist	115 (23.7)	179 (59.1)	149 (55.2)	443 (41.8)	<0.01
Gastrointestinal surgeon	102 (21.0)	77 (25.4)	79 (29.3)	258 (24.4)	0.04
Radiologist	401 (82.5)	206 (68.0)	183 (67.8)	798 (75.3)	<0.01
Pathologist	419 (86.2)	230 (75.9)	215 (79.6)	864 (81.6)	<0.01
Genetics MD	101 (20.8)	77 (25.4)	16 (5.9)	194 (18.3)	<0.01
Specialized nurse	246 (50.6)	52 (17.2)	41 (15.2)	339 (32.0)	<0.01
Radiation oncologist	372 (76.5)	177 (58.4)	180 (66.7)	729 (68.8)	<0.01

C: Univariable and multi-level logistic regression analyses showing the associations between income classification and survey responses on whether core membership criteria* are met during the multi-disciplinary team meetings									
		Multi-disciplinary team core membership criteria*		Univariable logistic regression analysis			Multi-level analysis corrected for country		
Income classification	Total	Yes	No	OR	95% CI	P value	OR	95% CI	P value
High	486	192 (39.5)	294 (60.5)	1			1		
Upper-middle	303	33 (10.9)	270 (89.1)	0.2	0.1 to 0.3	<0.001	0.1	0.1 to 0.3	<0.001
Low or lower-middle	270	24 (8.9)	246 (91.1)	0.2	0.1 to 0.2	<0.001	0.1	0.1 to 0.3	<0.001

*Multi-disciplinary team core membership criteria were met when the following healthcare workers were represented: gynecological cancer surgeon, a medical oncologist or gynecological oncologist who prescribes systemic therapy, a radiation oncologist, a pathologist, a radiologist, and a nurse.

Gynecological oncologists were the most constant members of the multi-disciplinary team (92%). A quarter of respondents did not have a radiologist as a member, and a fifth did not have a pathologist as a multi-disciplinary team member. One-third of respondents identified nurse incorporation. The proportions of team members differed significantly across income categories (Table 2B).

Multi-disciplinary team core membership criteria were defined as a gynecological cancer surgeon, a medical oncologist or gynecological oncologist who prescribes systemic therapy, a radiation oncologist, a pathologist, a radiologist, and a nurse. Only 40%, 11%, and 9% of the respondents from high-income, upper-middle, and lower-middle/low-income countries, respectively, responded that multi-disciplinary team core membership criteria were met. In addition, the odds of respondents being involved in multi-disciplinary teams that met core requirements were significantly lower if from a middle/low-income country than a high-income country (Table 2C).

#### Cytoreductive Surgery

Two-thirds of the respondents used ‘cytoreductive surgery’ to describe surgery for advanced ovarian cancer. Most respondents acknowledged ‘no macroscopic disease’ as the goal of surgery. However, these responses differed significantly across income categories, as a substantial proportion of the lower-middle/low-income respondents answered that ‘macroscopic disease <1 cm’ was the goal of surgery (Table 3A). Two-thirds of the respondents stated they would resect macroscopic disease only to achieve their goal of surgery (see Table 3B for the proportions stratified by income category).

**Table 3:** Survey results regarding the questions about the goals and components of cytoreductive surgery and the type of resections

A: Survey results regarding the question ‘What is your goal of surgery in cytoreductive surgery for advanced-stage ovarian cancer?’, stratified by income category					
	**High-income**	**Upper-middle income**	**Lower-middle and low-income**	**Total**	**P value (χ^2^**)
Macroscopic disease <1 cm	40 (8.2)	37 (12.2)	75 (27.8)	152 (14.4)	**<0.001**
Macroscopic disease <2.5 mm	10 (2.1)	17 (5.6)	23 (8.5)	50 (4.7)	
No macroscopic disease	427 (87.9)	248 (81.8)	166 (61.5)	841 (79.4)	
Other	9 (1.9)	1 (0.3)	6 (2.2)	16 (1.5)	
**B: Survey results regarding the question ‘In order to achieve your goal of surgery, what types of resections would you routinely perform?’, stratified by income category**					
	**High- income**	**Upper-middle income**	**Lower middle and low-income**	**Total**	**P value** **(χ^2^)**
Macroscopic disease only	359 (73.9)	195 (64.4)	160 (59.3)	714 (67.4)	**<0.001**
Macroscopic and possible microscopic disease	122 (25.1)	106 (35.0)	108 (40.0)	336 (31.7)	
Unable to answer	5 (1.0)	2 (0.6)	2 (0.7)	9 (0.9)	
**C: Survey results regarding the question ‘What surgical procedures are performed, if required, to achieve your goal of surgery? Multiple answers possible’, stratified by income category**					
**Resections**	**High-income** **Yes, n (%)**	**Upper-middle income** **Yes, n (%)**	**Lower-middle and low-income** **Yes, n (%)**	**Total** **Yes, n (%)**	**P value (χ** ^**2**^ **)**
Uterus/ tubes/ovaries and infra-colic omentum	473 (97.3)	286 (94.4)	263 (97.8)	1023 (96.6)	**0.040**
Large/small bowel	461 (94.9)	227 (74.9)	189 (70.0)	878 (82.9)	**<0.001**
Upper abdominal viscera/disease (spleen, liver, distal pancreas, disease at porta hepatis)	371 (76.3)	170 (56.1)	101 (37.4)	645 (60.9)	**<0.001**
Diaphragm stripping and/or resection	401 (82.5)	164 (54.1)	108 (40.0)	673 (63.6)	**<0.001**
Total peritonectomy	213 (43.8)	146 (48.2)	106 (39.3)	465 (43.9)	0.099
Other (free text)	Total yes: excision abnormal nodes 47 (4.4%), thoracic procedures 18 (1.7%), peritoneal ablation 1 (0.1%), partial peritonectomy 16 (1.5%)				Not analyzed

The types of resections routinely performed during cytoreductive surgery are shown in Table 3C. Up to 25% of respondents in high-income countries reported that upper abdominal procedures were not routinely incorporated in cytoreductive surgery. This rate increased as income category decreased, with up to 45% of surgeons in upper-middle-income countries and 60–65% of surgeons in lower-middle/low-income countries not routinely incorporating upper abdominal procedures in cytoreductive surgery. Bowel resections were less frequently performed during cytoreductive surgery in middle/low-income countries.

Disease scoring (Peritoneal Carcinomatosis Index, Fagotti score,^17^ other) was used by one-third of the respondents during surgery. Half of the respondents used residual disease scoring (Completeness of Cytoreduction score, other).

#### Main Barriers to Care

Responses to the main barriers of ovarian cancer care predominantly were patient, disease, and social factors, irrespective of income category (Table 4). Besides, high-income respondents stated that the main barriers were lack of surgical time/staff and patient preferences. Middle-income and low-income respondents additionally experienced treatment costs and lack of access to radiology/pathology/genetic services as main barriers. Lack of access to chemotherapy or other systemic agents was reported by one-third of lower-middle/low-income respondents (Table 4).

**Table 4:** Survey results regarding the question about the main barriers to ovarian cancer care

Main barriers	Total Yes, n (%)	High- income Yes, n (%)	Upper-middle income Yes, n (%)	Lower-middle/ low-income Yes, n (%)	P value (χ*^2^*)
Patient factors (elderly, frail, medical co-morbidities)	662 (62.5)	338 (69.5)	165 (54.5)	159 (58.9)	**<0.01**
Disease factors (late stage disease/large tumor burden at diagnosis)	735 (69.4)	288 (59.3)	219 (72.3)	228 (84.4)	**<0.01**
Diagnostic factors (lack of access to diagnostic procedures: radiology, pathology)	219 (20.7)	24 (4.9)	91 (30.0)	104 (38.5)	**<0.01**
Diagnostic factors (lack of expertise: radiologists, pathologists)	140 (13.2)	17 (3.5)	51 (16.8)	72 (26.7)	**<0.01**
Treatment factors: lack of surgical expertise	200 (18.9)	45 (9.3)	69 (22.8)	86 (31.9)	**<0.01**
Treatment factors: lack of surgical time, surgical equipment, support staff	276 (26.1)	91 (18.7)	88 (29.0)	97 (35.9)	**<0.01**
Treatment factors: lack of medical oncology expertise	89 (8.4)	16 (3.3)	29 (9.6)	44 (16.3)	**<0.01**
Treatment factors: lack of access to chemotherapy or systemic agents	198 (18.7)	27 (5.6)	81 (26.7)	90 (33.3)	**<0.01**
Peri-operative care (lack of ICU beds, critical care staff, equipment)	239 (22.6)	65 (13.4)	77 (25.4)	97 (35.9)	**<0.01**
Genetic service access (lack of resources for BRCA/HRD testing)	371 (35.0)	39 (8.0)	153 (50.5)	179 (66.3)	**<0.01**
Social factors (patient travel, distance, social support systems)	443 (41.8)	120 (24.7)	143 (47.2)	180 (66.7)	**<0.01**
Cost of treatment	365 (34.5)	57 (11.7)	121 (39.9)	187 (69.3)	**<0.01**
Patient preference (for no treatment or alternative treatments)	223 (21.1)	102 (21.0)	38 (12.5)	83 (30.7)	**<0.01**

## Discussion

### Summary of Main Results

The current study showed that respondents from lower-middle/low-income countries were significantly less likely to undertake extensive resections during cytoreductive surgery than respondents from high-income countries. However, it should be noted that almost 25% of the high-income respondents stated that upper abdominal surgery was not routinely performed during cytoreductive surgery. Significantly fewer regional networks, cancer registries, quality registries, and patient advocacy groups existed in lower-middle/low-income countries. However, there is also scope for improvement in using these components of optimal care in high/upper-middle-income countries. Finally, disease scoring systems were used by a minority of the respondents.

The main barriers to optimal care were patient factors, disease factors, and social factors, irrespective of the income category. Besides, high-income respondents stated that the main barriers were lack of surgical time/staff and patient preferences. Middle-income and low-income respondents additionally experienced treatment costs and lack of access to radiology/pathology/genetic services as main barriers. Lack of access to chemotherapy or systemic agents was reported by one-third of lower-middle/low-income respondents.

### Results in the Context of Published Literature

Recently, a similar study was published by Norell et al that reported results from a survey on treatment guidelines and patterns of care for ovarian cancer in seven high-income countries.^7^ In line with the results of the current study, international differences were found in whether extensive resections were performed during cytoreductive surgery (or willingness to undertake extensive surgeries). Moreover, Norell et al found an association between survival and willingness to undertake extensive surgery. Therefore, the poorer survival for patients with ovarian cancer in certain countries could partly be explained by the fact that extensive cytoreductive surgeries are less frequently performed in certain regions.

Focusing on the barriers to optimal care, the results of Norell et al overlap with the results of the current study. In both studies, high-income respondents stated that treatment costs and lack of surgical time and staff were the main barriers. Additionally, the study by Norell et al found a lack of treatment monitoring (auditing), and the current study found patient, disease, and social factors as main barriers. The differences in these results could be explained by the fact that Norell et al did not include the patient and disease factors as answer options and lack of auditing was not an answer option in the current study. However, we found that a substantial proportion stated that their hospital did not contribute to a cancer/quality registry. The main barriers for physicians treating ovarian cancer in middle/low-income countries have not yet been described.

The causes of disparities in cancer care and the potential solutions to bridge the gap have been described by Abuali et al (data from the USA, not focused on ovarian cancer).^18^ The authors found that access to care (rural disadvantages, economic limitations), bias in care (variation in care quality), financial toxicity (drug costs, healthcare insurance costs), and access to clinical trials (under-representation of ethnic and racial minorities) were the most important causes for disparities. These results overlap with the results of the current study, as the surveyed physicians stated that, besides other barriers, social factors, treatment costs, and lack of access to diagnostics and systemic agents were the main barriers to optimal care. Potential solutions were described by Abuali et al to bridge the care gap: partnering with community leaders and patient advocacy groups, more equitable research funding allocation, improved access to clinical trials for ethnic and racial minorities, and diversification of the workforce. Whether these potential solutions could help to diminish the disparities in ovarian cancer care should be discussed with regional representatives.

Overall, the literature on the organization of ovarian cancer care in middle- and low-income countries is scarce. Despite a well-recognized and growing need for access to cancer surgery in middle- and low-income countries, there has been minimal discourse in the global health literature regarding the state of surgical care or strategies to improve it.^19^ International disparities in patient experience of ovarian cancer care have been described, and significant heterogeneity in patient experience between countries was observed.^20^ The patients’ perspectives were outside the scope of the current study. However, it is essential to consider the patients’ perspectives when strategies are developed to improve ovarian cancer care.

### Strengths and Weaknesses

The current report describes the largest cohort in expert opinion surveys relating to ovarian cancer care in gynecological oncology published to date. In addition, substantial proportions of the respondents worked in middle/low-income countries while the current literature only describes high-income countries. Therefore, the results of the current study give a unique insight into the challenges physicians face in these regions. Another strength is that international experts in ovarian cancer care rigorously defined the questions. In addition, the survey was sent to a wide global network and written in multiple languages, improving the accuracy of the data collected.

There are certain weaknesses in the current study design. First, selection bias is inevitable in survey studies. The respondents were predominantly contacted through certain networks, therefore physicians not linked to these networks are under-reported in the survey. However, during an interim analysis of the respondent characteristics, gaps in represented regions and countries were identified and physicians outside the networks were contacted. Second, non-responses were not tracked and therefore the extent of the selection bias could not clearly be described. Third, the current answers to the survey questions may not exactly reflect the actual patterns of care. The more reliable standard for evaluating patterns of care is through clinical quality registries. However, most countries lack such registries and data from existing registries are often unavailable. Last, respondents were stratified into income categories, but countries inside the different income categories are not always comparable and, even within countries, there are vast differences.

### Implications for Practice and Future Research

The findings of the current study have important implications for the global ovarian cancer care organization. Further research is necessary, specifically focusing on regional data analysis and qualitative analysis of the open-ended responses. The implications of the study suggest the need for implementing several key actions. First, enhancing accessibility to chemotherapy and other systemic agents, especially in lower-middle/low-income countries, is crucial, and involving pharmaceutical companies in achieving this objective is essential. Second, improving the availability of pathology, radiology, and genetic services in middle- and low-income countries is important, and regional representatives will be engaged in the Global Equality in Ovarian Cancer Care project to address this issue. Third, enabling gynecological cancer surgeons and gynecologists across all income categories to perform extensive resections during cytoreductive surgery should be prioritized, with support from the International Gynecologic Cancer Society in training and collaboration with regional representatives. Fourth, increasing the utilization of cancer registries, quality registries, and patient advocacy groups globally is recommended, although implementation may pose challenges in middle- and low-income countries. The International Gynecologic Cancer Society will engage existing national cancer and quality registries to encourage collaboration and information sharing on initiating registries, while emphasizing the importance of local political support for successful registry launch. Last, efforts to promote the use of disease scoring systems will be addressed at the upcoming global meeting of the International Gynecologic Cancer Society in Seoul in November 2023.

## Conclusions

Global disparities exist in the organization of care for patients with ovarian cancer. The current study gives an insight into the challenges that physicians face around the globe. Additional analyses will be performed on the survey data. These analyses will be discussed with the regional representatives to develop regional or country-specific recommendations. Ultimately, this project aims to implement these recommendations and improve care to diminish the survival disparities for patients with ovarian cancer.

## Data Availability

Data are available upon reasonable request.
