# Supplementary material for: Exploring international differences in ovarian cancer care: a survey report on global patterns of care, current practices, and barriers
Source: Int J Gynecol Cancer. 2023 Aug 17;33(10):1612–20. doi: 10.1136/ijgc-2023-004563 (PMC10579489; doi:10.1136/ijgc-2023-004563)
Supplement: Supplementary data [file ijgc-2023-004563supp001.pdf]

Supplementary Table 1: Global Equality in Ovarian Cancer Care Survey questions and answer options.

**Survey Question**	**Answer options**
Section 1: Respondent characteristics	
Q1: In which country do you work?	Multiple choice: country list
Q2: In which region (state, province, region, or jurisdiction) do you work?	Free text
Q3: What is the name of the main hospital and/or university where you currently work (clinical work/ research)?	Free text
Q4: In what ZIP/postal code is your hospital/clinical practice located (for non-US citizens)?	Free text
Q5: In what ZIP code is your hospital/clinical practice located (for US citizens)?	Free text
Q6: What best describes your profession or specialty?	Multiple choice:<br>x Gynecological oncologist (surgery and systemic therapy)<br>x Gynecological oncology surgeon<br>x Surgical oncologist<br>x Medical oncologist<br>x Radiation oncologist<br>x Obstetrician/gynecologist<br>x Other (please specify)
Section 2: Organization of national/regional healthcare	
Q7: What proportions of healthcare for ovarian cancer is provided in public and private practice in your country/ region/ state/ province/ jurisdiction?	Percentage
Q8: Which, if any, international guidelines do you use to inform your ovarian cancer treatment recommendations?	Multiple choice:<br>x NCCN<br>x ESGO<br>x ESMO<br>x Other (please specify)
Q9: Which, if any, national guidelines do you use to inform your ovarian cancer treatment recommendations?	Free text
Q10: Could you estimate the proportion of patients treated according to these guidelines?	Multiple choice:<br>x <20%<br>x 20-75%<br>x >75%
Q11: Are there any regional/national mandatory requirements for hospitals to treat ovarian cancer? If so, which requirements?	Multiple choice:<br>x No<br>x Yes, please specify
Q12: If yes (Q11), for which entity does this apply (multiple answers are possible)	Multiple choice:<br>x My hospital/clinical practice<br>x My state<br>x My province<br>x My region<br>x My jurisdiction<br>x My country
Q13: Does your hospital contribute to a regional/national cancer registry, where the incidence of ovarian cancer is registered?	Multiple choice:<br>x Yes, national<br>x Yes, regional<br>x No
Q14: Does your hospital contribute to a quality registry, where the quality of healthcare of ovarian cancer is registered?	Multiple choice:<br>x Yes, with benchmarking<br>x Yes, without benchmarking<br>x No
Q15: Does your region/country have an active patient advocacy group?	Multiple choice:<br>x Yes<br>x No
Q16: Is there a gynecological oncology (surgical) training program in your region/country?	Multiple choice:<br>x Yes, an accredited program<br>x Yes, a non-accredited program<br>x No

Algera MD, *et al. Int J Gynecol Cancer* 2023; 33:1612–1620. doi: 10.1136/ijgc-2023-004563

Q17: Are there any regional networks in agreements concerning multidisciplinary teams (MDT), referral, and location of surgical procedures listed between referring hospitals and ovarian cancer centers (or gynecological oncology) in your region/country?	Multiple choice:<br>x No<br>x Yes, please specify
Q18: Are there any regional (gynecological) oncology centers where the healthcare for ovarian cancer is concentrated?	Multiple choice:<br>x Yes<br>x No
Section 3: Organization of healthcare in the individual hospitals	
Q19: What term do you use to describe surgery for advanced-stage ovarian cancer in your hospital?	Multiple choice:<br>x Cytoreductive surgery<br>x Debulking surgery
Q20: Do you use any standardized scoring systems to document the volume of disease present during cytoreductive surgery for advanced-stage ovarian cancer?	Multiple choice:<br>x Yes, peritoneal carcinomatosis index (PCI)<br>x Yes, Fagotti score [18]<br>x Yes, other please specify<br>x No
Q21: What is your goal of surgery in cytoreductive surgery for advanced-stage ovarian cancer?	Multiple choice:<br>x No macroscopic disease<br>x Macroscopic disease present <2.5 mm<br>x Macroscopic disease present <1.0 cm<br>x Other, please specify
Q22: In order to achieve your goal of surgery, what types of resections would you routinely perform?	Multiple choice:<br>x Resection of macroscopic disease<br>x Resection of microscopic and macroscopic disease<br>x Other, please specify
Q23: What surgical procedures are performed, if required, to achieve your goal of surgery?	Multiple choice, multiple answers:<br>x resection of uterus/tubes/ovaries/ infra-colic omentum<br>x resection of large/small bowel<br>x resection of upper abdominal disease<br>x diaphragm stripping and/or resection<br>x total peritonectomy<br>x Other, please specify
Q24: Who performs surgical procedures for ovarian cancer at your hospital?	Multiple choice:<br>x Obstetrician/gynecologist<br>x Gynecological oncologist<br>x Surgical oncologist
Q25: How do you document residual disease in your hospital?	Multiple choice:<br>x Completeness of cytoreduction score<br>x Other score<br>x No
Q26: How many cytoreductive surgeries are performed annually in your hospital?	Multiple choice:<br>x 0 – 20<br>x 21 – 40<br>x 41 – 60<br>x 61 – 80<br>x 81 – 100<br>x 101 – 150<br>x >150
Q27: Is it standard of care to discuss patients with ovarian cancer in a MDT in your hospital?	Multiple choice:<br>x Yes, for all patients<br>x Yes, for selected patients<br>x No
Q28: Which specialists are in the MDT?	Multiple choice, multiple answers:<br>x Gynecological oncologist<br>x Medical oncologist<br>x Surgical oncologist<br>x Gastro-intestinal surgeon<br>x Radiologist<br>x Pathologist<br>x Genetic MD<br>x Specialized nurse<br>x Radiation oncologist<br>x Other, please specify:

Q29: What are the main barriers to optimal ovarian cancer care in your hospital, region, or country?	Multiple choice:<br>x Patient factors (elderly, frail, medical comorbidities)<br>x Disease factors (late stage disease / large tumor burden at diagnosis)<br>x Diagnostic factors (lack of access to diagnostic procedures: radiology, pathology)<br>x Diagnostic factors (lack of expertise: radiologists, pathologists)<br>x Treatment factors - lack of surgical expertise<br>x Treatment factors - lack of surgical time, surgical equipment, support staff<br>x Treatment factors - lack of medical oncology expertise<br>x Treatment factors - lack of access to chemotherapy or systemic agents<br>x Perioperative care (lack of ICU beds, critical care staff, equipment)<br>x Genetic service access (lack of resources for BRCA/HRD testing)<br>x Social factors (patient travel, distance, social support systems)<br>x Cost of treatment<br>x Patient preference (for no treatment or alternative treatments)
Q30: Comments on Q29.	Free text

Algera MD, *et al. Int J Gynecol Cancer* 2023; 33:1612–1620. doi: 10.1136/ijgc-2023-004563

Supplementary Table 2: Number of respondents per country (and continent).

**Africa** **N=93**		**Asia/ Middle East** **N=323**		**Europe** **N=249**		**North / Middle America, Caribbean** **N=205**		**Oceania** **N=22**		**South America** **N=167**	
Algeria	N=1	Bahrein	N=2	Albania	N=2	Barbados	N=1	Australia	N=17	Argentina	N=29
Botswana	N=1	Bangladesh	N=12	Armenia	N=1	Canada	N=15	Fiji	N=1	Bolivia	N=1
Cameroon	N=2	Bhutan	N=1	Austria	N=6	Costa R.	N=7	N. Zealand	N=4	Brazil	N=76
D.R. Congo	N=1	Cambodia	N=1	Belarus	N=5	El Salvador	N=4			Chile	N=15
Egypt	N=7	China	N=21	Belgium	N=3	Guatemala	N=14			Colombia	N=15
Ethiopia	N=8	India	N=74	Bulgaria	N=1	Haiti	N=6			Ecuador	N=4
Ghana	N=5	Indonesia	N=22	Croatia	N=2	Honduras	N=1			Paraguay	N=1
Kenya	N=9	Iran	N=2	Czech R.	N=4	Jamaica	N=4			Peru	N=11
Libya	N=1	Iraq	N=1	Denmark	N=1	Mexico	N=45			Uruguay	N=6
Malawi	N=1	Israel	N=7	Estonia	N=2	Nicaragua	N=4			Venezuela	N=9
Morocco	N=1	Japan	N=49	Finland	N=3	Panama	N=2				
Mozambique	N=4	Kazakhstan	N=8	France	N=6	Bahamas	N=3				
Namibia	N=1	Kuwait	N=1	Georgia	N=1	Trinidad & T.	N=1				
Nigeria	N=19	Kyrgyzstan	N=1	Germany	N=10	U.S.A.	N=98				
Rwanda	N=1	Lebanon	N=3	Greece	N=13						
Senegal	N=1	Malaysia	N=16	Hungary	N=1						
South Africa	N=13	Mongolia	N=2	Ireland	N=6						
Sudan	N=3	Myanmar	N=2	Italy	N=34						
Tunisia	N=5	Nepal	N=8	Latvia	N=1						
Uganda	N=5	Pakistan	N=5	Lithuania	N=2						
Zambia	N=4	Philippines	N=23	Macedonia	N=2						
		Qatar	N=3	Netherlands	N=23						
		Saudi A.	N=2	Norway	N=2						
		Singapore	N=3	Poland	N=4						
		S. Korea	N=34	Portugal	N=3						
		Taiwan	N=1	Rep. Serbia	N=4						
		Tajikistan	N=1	Romania	N=4						
		Thailand	N=9	Russia	N=7						
		U.A.E.	N=1	Slovakia	N=3						
		Uzbekistan	N=4	Slovenia	N=2						
		Vietnam	N=4	Spain	N=11						
				Sweden	N=9						
				Switzerland	N=4						
				Turkey	N=14						
				Ukraine	N=6						
				U.K.	N=47						
